# A Fatal Triad of Myeloradiculitis, Multifocal Cerebral Infarction, and Central Retinal Artery Occlusion Revealing Occult Giant Cell Arteritis

**DOI:** 10.7759/cureus.117809

**Published:** 2026-10-12

**Authors:** Safaa Zahlan, Douaa Elmejdoubi, Oumayma Azzou, Nissrine Louhab

**Affiliations:** 1 Neurology, Mohammed VI University Hospital, Marrakech, MAR; 2 Neurology, Cadi Ayyad University, Mohammed VI University Hospital, Marrakech, MAR; 3 Neurology, Faculty of Medicine and Pharmacy, Cadi Ayyad University, Mohammed VI University Hospital, Marrakech, MAR

**Keywords:** central retinal artery occlusion, giant cell arteritis, glucocorticoid therapy, large-vessel vasculitis, multifocal cerebral infarction, myeloradiculitis, sepsis

## Abstract

Giant cell arteritis (GCA) is the most common primary systemic vasculitis in adults over 50 years of age and classically presents with cranial symptoms such as headache, jaw claudication, and scalp tenderness. Spinal cord involvement is exceedingly rare and easily mistaken for degenerative spinal disease, particularly when headache, jaw claudication, and scalp tenderness are absent. We report the case of a 61-year-old woman whose illness evolved over several months, beginning with a lower-limb deficit and sciatica misdiagnosed as a disc herniation and treated surgically without improvement. Only when a contralateral deficit, a sensory level, and sphincter dysfunction emerged was a thoracic myeloradiculitis recognized on MRI. Days before admission, an acute monocular visual loss and a new upper-limb deficit revealed a more extensive process, and biopsy-confirmed GCA with large-vessel involvement and multifocal cerebral lesions was ultimately diagnosed. Despite an initial partial response to high-dose glucocorticoids, the patient died of septic shock before advanced imaging for disease-extent assessment could be performed, owing to local resource constraints. This case illustrates the diagnostic pitfalls of spinal-onset GCA without headache, jaw claudication, or scalp tenderness, and the infectious risk of high-dose glucocorticoid therapy in severe large-vessel disease.

## Introduction

Giant cell arteritis (GCA) is a granulomatous vasculitis of large and medium-sized vessels, occurring almost exclusively after the age of 50, with a female predominance. Its clinical spectrum is classically dominated by cranial manifestations - new-onset temporal headache, jaw claudication, scalp tenderness, and abrupt visual loss - reflecting the vasculitic predilection for the extracranial branches of the carotid arteries [1]. GCA is, however, increasingly recognized as a systemic large-vessel disease, with aortic and supra-aortic involvement exposing patients to a distinct set of complications, including aortic aneurysm, carotid or vertebral artery occlusion, and cerebral infarction.

Spinal cord involvement remains exceptional and probably underrecognized, reported in only a limited number of cases worldwide. Reported mechanisms include spinal cord ischemia from occlusive vasculitis of the radicular or spinal arteries arising from an inflamed, ectatic aorta [2,3]; whether inflammation of the spinal cord and its nerve roots (myeloradiculitis) can also occur remains uncertain.

We report a fatal case of GCA that began with a thoracic myeloradiculitis mistaken for degenerative disc disease, in the absence of headache, jaw claudication, or scalp tenderness, and evolved through multifocal cerebral lesions of probable vascular origin, central retinal artery occlusion (CRAO), and fatal sepsis, in the context of biopsy-confirmed GCA with large-vessel involvement.

## Case presentation

A 61-year-old woman with a three-month history of treated hypertension was admitted with a several-month history of progressive myelopathic and multifocal neurological deficits.

Her symptoms began several months earlier with left lower-limb weakness and sciatica, interpreted as a lumbar disc herniation. Lumbar MRI, performed externally approximately three to four months before admission, showed advanced multilevel disc-osteoarthritis with severe canal stenosis (Schizas grade C) at L4-L5 and L5-S1 and bilateral conflict with the L5 and S1 root emergences, moderate narrowing at L3-L4, and a normal conus medullaris; only the radiology report was available to the authors. She subsequently underwent an L4-L5 laminectomy without any improvement. No electrophysiological study was performed before admission.

Over the following months, her left lower-limb deficit progressed to a complete motor loss that did not respond to rehabilitation, and - the reason for the current hospitalization - she developed a new deficit of the contralateral (right) lower limb with urinary retention. Four days before admission, she additionally developed a right upper-limb weakness together with redness of the left eye and a rapidly progressive loss of vision in that eye. Throughout this period, she reported nocturnal fever, anorexia, and unquantified weight loss.

On admission, she was alert (Glasgow Coma Scale 15/15) and clinically stable from a hemodynamic and respiratory standpoint, with redness of the left eye and bilaterally diminished, indurated temporal pulses; blood pressure was asymmetric between the two arms (104/68 mmHg on the left, 92/61 mmHg on the right). Neurological examination showed that she could neither stand nor walk; she was able to hold a Barré position but not a Mingazzini position [4]. Segmental motor testing (Medical Research Council (MRC) scale [5]) showed a right upper limb graded 4/5 (proximal and distal), a normal left upper limb (5/5), and flaccid paralysis of both lower limbs (0/5, proximal and distal), with hypotonia confined to the lower limbs. Deep tendon reflexes were preserved and symmetric in the upper limbs but abolished in the lower limbs; the plantar responses were indifferent bilaterally, and Hoffmann's sign was absent. There was right-sided dysmetria on finger-to-nose testing. All sensory modalities - superficial, deep, and thermoalgesic (pain and temperature) - were impaired in the lower limbs, with a sensory level at T10 (umbilical dermatome). Cranial nerve examination was notable for severe visual loss in the left eye (counting-fingers vision), with fundoscopy showing an occlusion of the central retinal artery; the remaining cranial nerves were normal, as was higher cortical function. Altogether, the clinical picture combined a myelopathic (spinal cord) syndrome with a right hemicerebellar syndrome, indicating a multifocal neurological process.

On admission, the complete blood count showed hemoglobin 11.9 g/dL, leukocytes 5.50 × 10⁹/L (neutrophils 3.69 × 10⁹/L, lymphocytes 1.16 × 10⁹/L), and platelets 277 × 10⁹/L. C-reactive protein was 56.9 mg/L, the erythrocyte sedimentation rate 48 mm at the first hour and 76 mm at the second hour, and procalcitonin 0.04 ng/mL. Serum sodium was 134 mmol/L and potassium 4.10 mmol/L, with urea 0.39 g/L and creatinine 6.15 mg/L (Table 1). Cerebrospinal fluid (CSF) analysis showed an elevated protein level (1.64 g/L) with a mildly reduced glucose (0.32 g/L, in the absence of a paired serum glucose to calculate a CSF/serum ratio); GeneXpert testing for tuberculosis in the CSF and Lyme serology in the CSF were both negative. Oligoclonal band analysis of paired CSF and serum samples showed a type 3 pattern. Serum angiotensin-converting enzyme was normal, and serologies for syphilis, HIV, and Lyme disease were all negative. Antineutrophil cytoplasmic antibodies (c-ANCA and p-ANCA) and anti-double-stranded DNA antibodies were negative (Table 2).

**Table 1:** Quantitative laboratory findings, with reference ranges Late values: electrolytes and C-reactive protein at the septic episode; blood count, the last available in intensive care; CSF: cerebrospinal fluid

Parameter	Admission	Late (septic episode/intensive care)	Reference range
Hemoglobin (g/dL)	11.9	10.1	12–14
Leukocytes (× 10⁹/L)	5.50	3.46	4–10
Neutrophils (× 10⁹/L)	3.69	3.34	2–7
Lymphocytes (× 10⁹/L)	1.16	0.07	1–4
Platelets (× 10⁹/L)	277	32	150–450
C-reactive protein (mg/L)	56.9	8	<5
Erythrocyte sedimentation rate, 1st hour (mm/h)	48	Not repeated	<20
Procalcitonin (ng/mL)	0.04	Requested, not processed	<0.05
Serum sodium (mmol/L)	134	119	135–145
Serum potassium (mmol/L)	4.10	2.7	3.5–5.0
CSF protein (g/L)	1.64	-	0.15–0.45

**Table 2:** Qualitative laboratory, cerebrospinal fluid, and histological findings CSF: cerebrospinal fluid; c-ANCA: cytoplasmic antineutrophil cytoplasmic antibody; p-ANCA: perinuclear antineutrophil cytoplasmic antibody

Parameter	Result
Complete blood count	Normal
CSF GeneXpert (tuberculosis)	Negative
CSF Lyme serology	Negative
CSF/serum oligoclonal bands	Type 3 pattern
Serum angiotensin-converting enzyme	Normal
Serum syphilis serology	Negative
Serum HIV serology	Negative
Serum Lyme serology	Negative
c-ANCA	Negative
p-ANCA	Negative
Anti-double-stranded DNA antibodies	Negative
Blood culture (aerobic and anaerobic)	*Streptococcus* species (Gram-positive cocci)
Temporal artery biopsy (histology)	Lympho-histiocytic infiltrate with periadventitial cuffing of vasa vasorum, intimal hyperplasia, elastic fiber fragmentation; consistent with temporal arteritis
Minor salivary gland biopsy (histology)	Unremarkable; no granulomatous inflammation or lymphoproliferative features

Spinal MRI showed swelling of the thoracic cord at the T11-T12 level and of the conus medullaris (Figure 1), with contrast enhancement, which was interpreted as consistent with an inflammatory myeloradiculitis; an ischemic mechanism could not be excluded. Brain MRI showed FLAIR hyperintensities in the left frontal cortico-subcortical region, the right parietal and occipital regions, the right cerebellar white matter, and the bulbar and cervical cord, the latter without enhancement after gadolinium (Figure 2). The right parietal lesion showed diffusion-weighted hyperintense foci with minimal hemorrhagic remodeling. The parietal and cerebellar lesions were T1-hyperintense, and ADC values were increased. The radiologists considered the pattern in favor of a vascular origin. Transthoracic echocardiography showed a preserved left ventricular ejection fraction (65%) with a dilated ascending aorta. Supra-aortic trunk Doppler ultrasound showed dampened flow with an acceleration signal in the right internal carotid artery, and CT angiography confirmed a total occlusion of the right internal carotid artery (Figure 3), with a patent left internal carotid artery, together with ectasia of the aorta (Figure 4).

**Figure 1:**
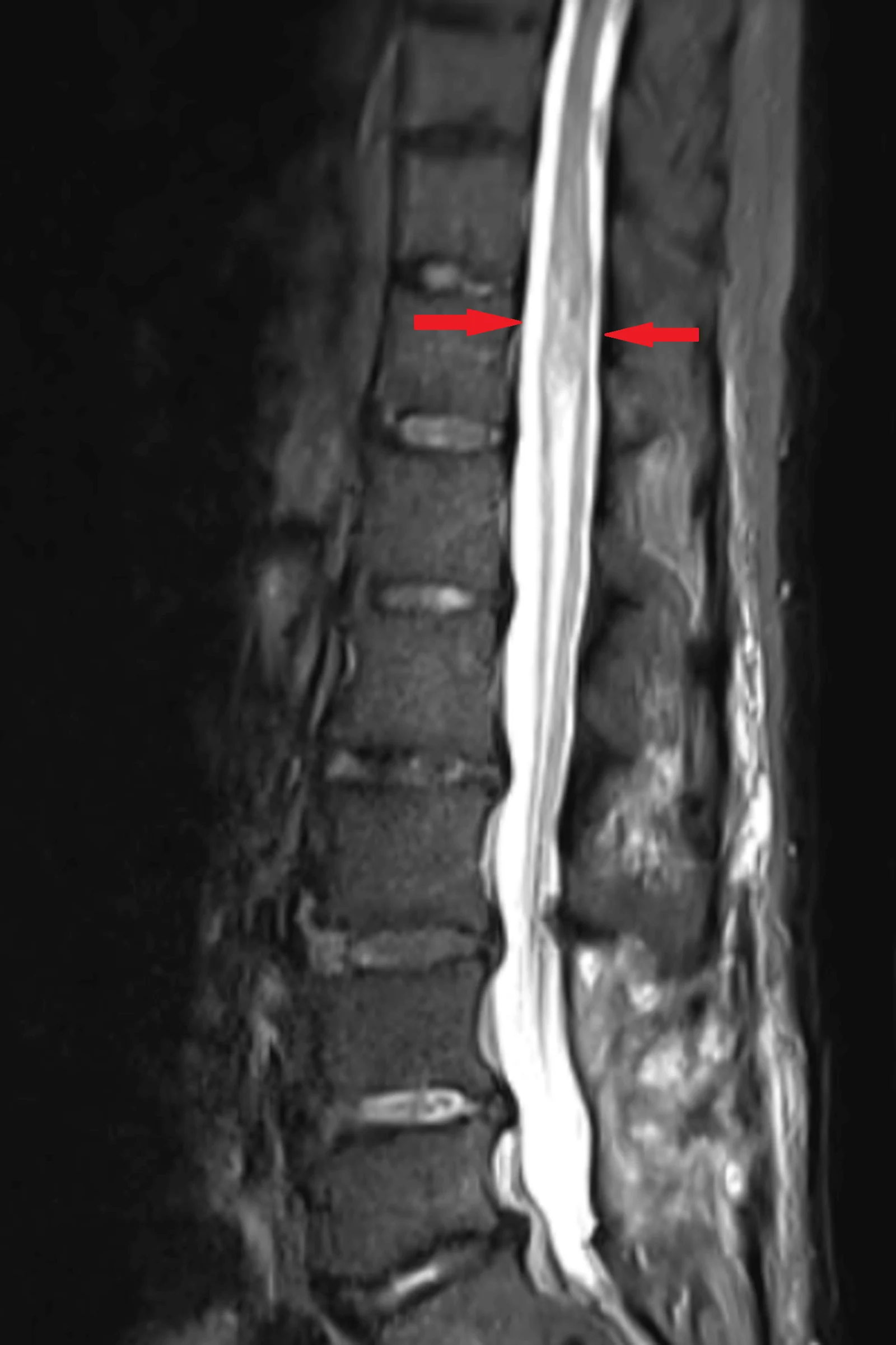
Sagittal T2-weighted spinal MRI showing swelling of the thoracic cord and conus medullaris (arrows).

**Figure 2:**
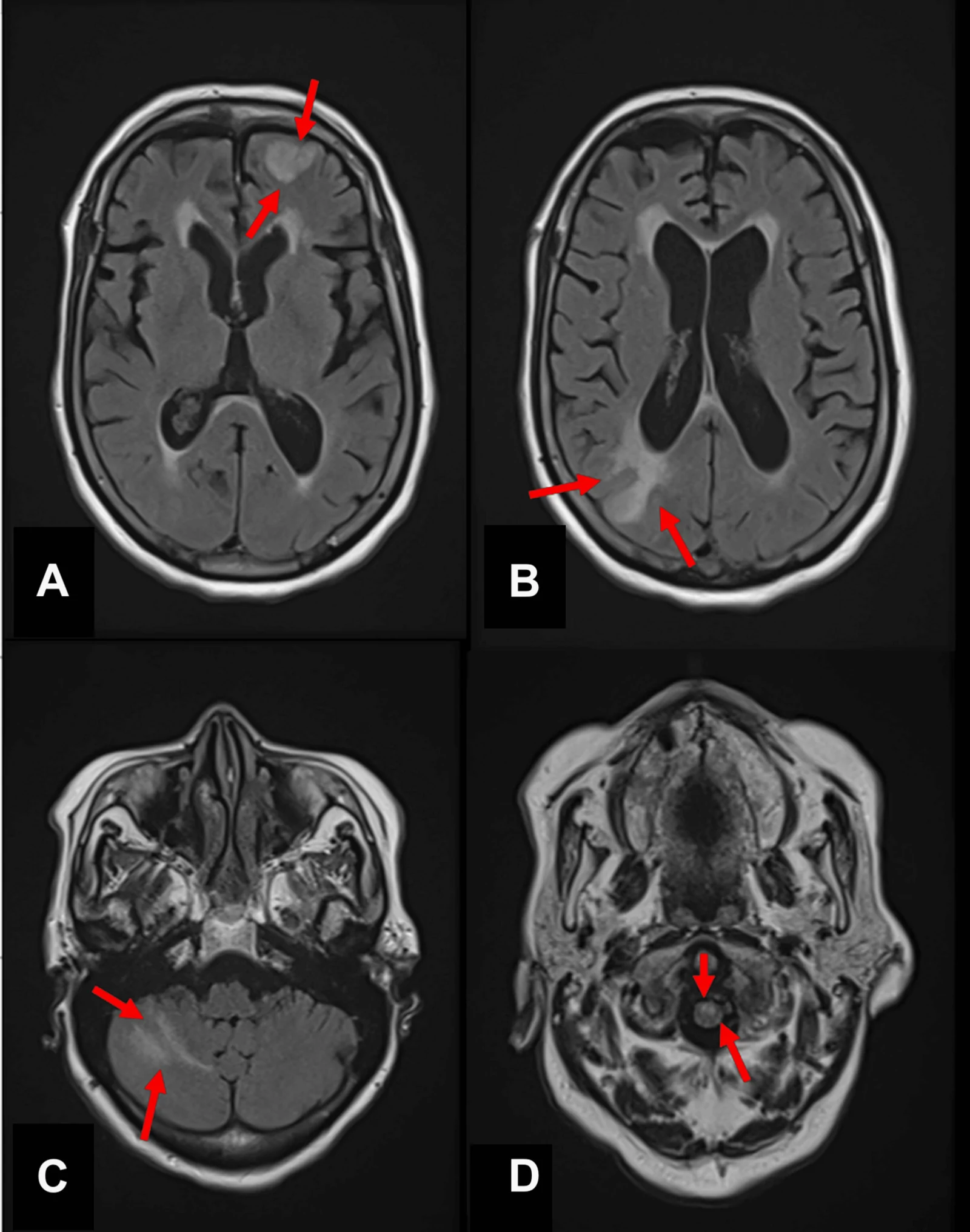
Axial FLAIR brain MRI. (A) Hyperintensity of the left frontal white matter, both subcortical and periventricular in location (arrows). (B) Slightly higher axial slice showing a right parieto-occipital periventricular hyperintensity (arrows). (C) Axial image at the level of the posterior fossa demonstrating a hyperintensity within the right cerebellar hemisphere (arrows). (D) Axial image at the level of the bulbo-medullary junction showing bilateral symmetric FLAIR-hyperintense foci involving the ventral cord (arrow). FLAIR MRI: fluid-attenuated inversion recovery magnetic resonance imaging

**Figure 3:**
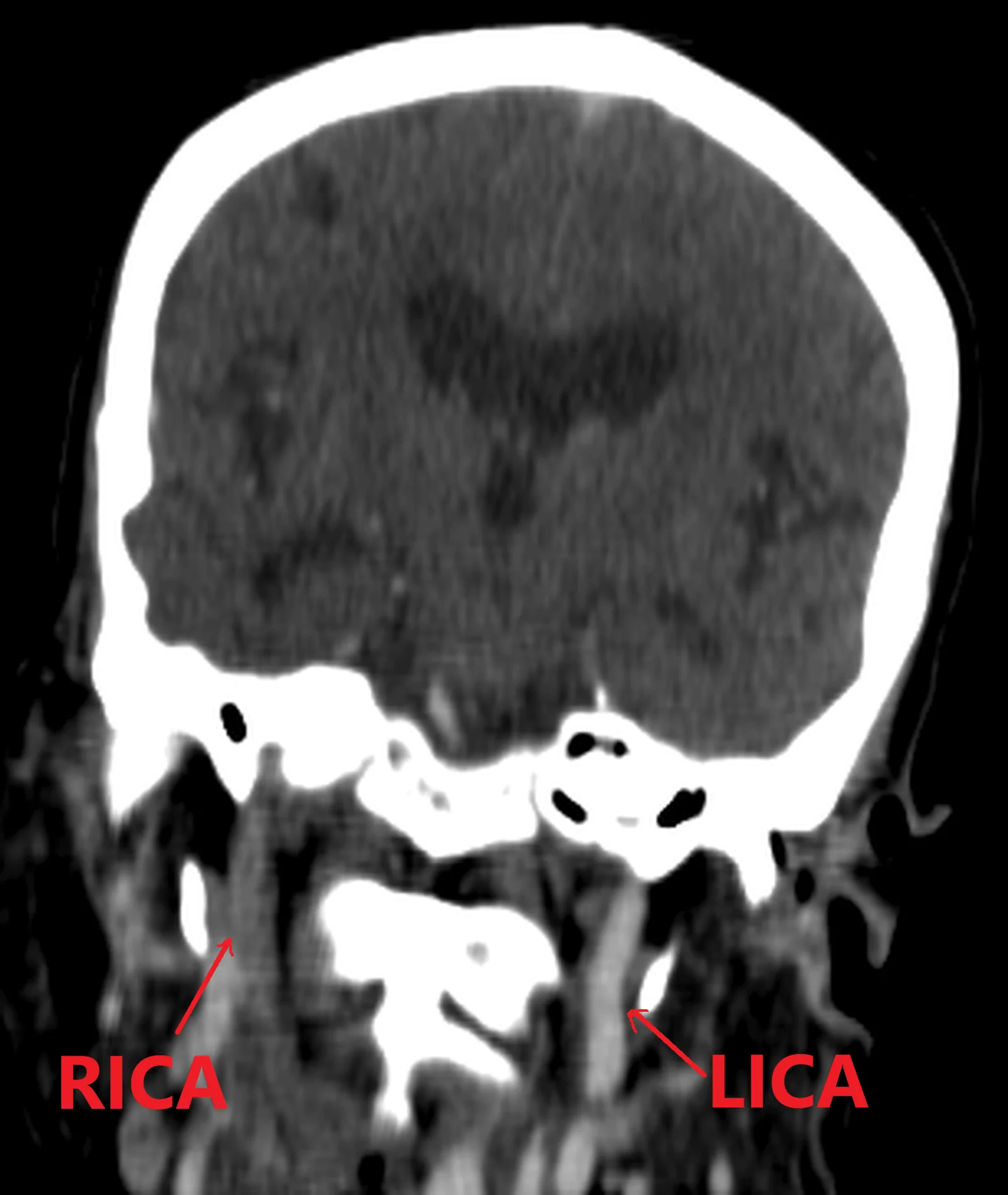
Coronal contrast-enhanced neck CT reformat showing the right internal carotid artery (RICA, arrow), faintly opacified and consistent with occlusion, and the left internal carotid artery (LICA, arrow), well opacified and patent for comparison.

**Figure 4:**
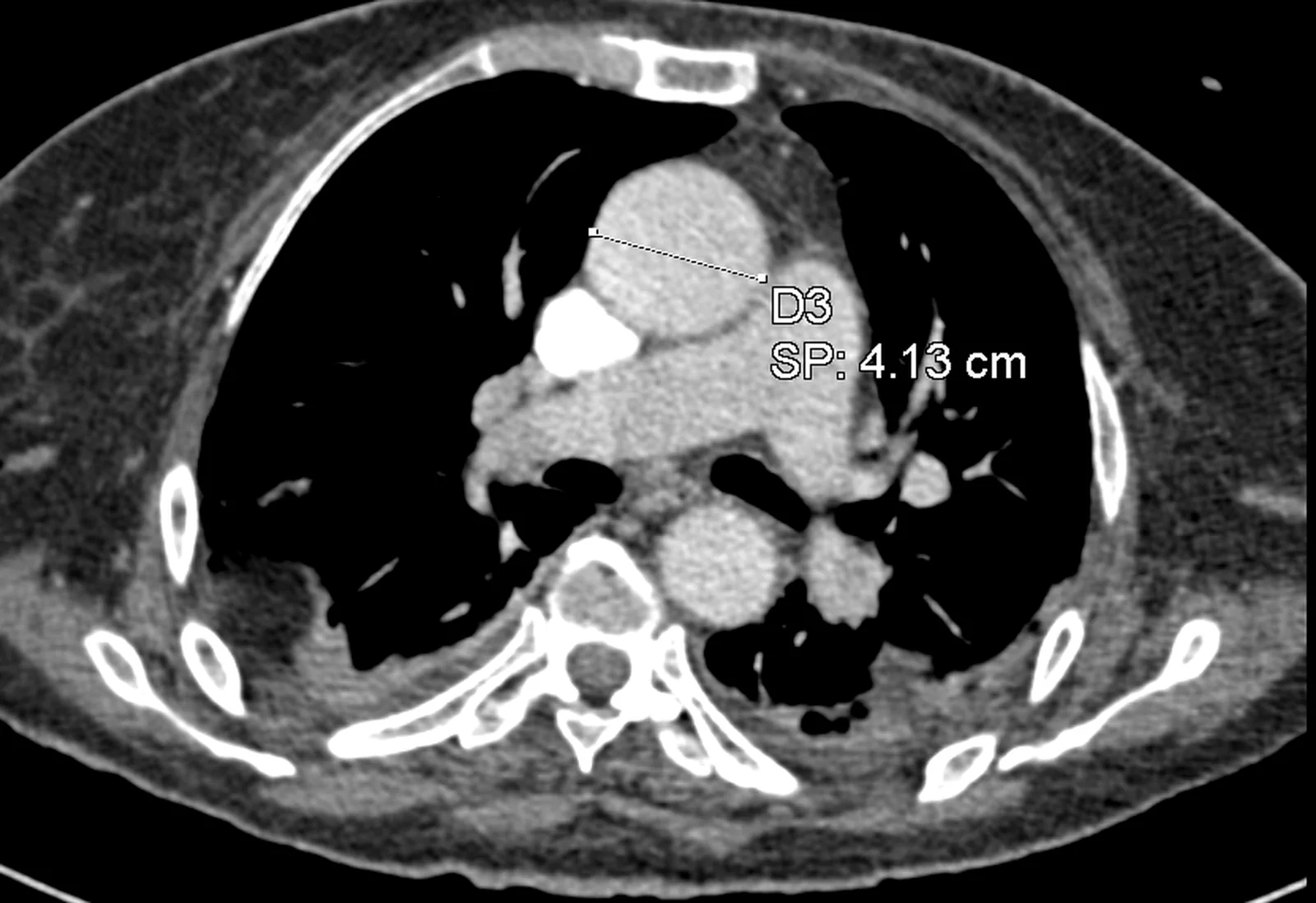
Axial contrast-enhanced chest CT (vascular/mediastinal window) showing ectasia of the ascending aorta, measuring 4.13 cm in diameter (dashed line), consistent with the aortic dilation described on CT angiography.

Treatment was initiated with intravenous methylprednisolone pulses (1 g/day for five days) together with third-generation cephalosporin coverage and physical rehabilitation, with improvement in right upper-limb strength. An internal medicine opinion was requested.

A temporal artery biopsy was undertaken because of the combination of a systemic inflammatory syndrome, bilaterally diminished and indurated temporal pulses, CRAO, and large-vessel involvement on imaging. A minor (accessory) salivary gland biopsy was performed at the same time to screen for granulomatous (e.g., sarcoidosis) and lymphoproliferative mimics of systemic vasculitis. The right temporal artery specimen (a 2.5 cm segment) showed a lympho-histiocytic inflammatory infiltrate predominating in the adventitia, with periadventitial cuffing of the vasa vasorum, intimal hyperplasia, and fragmentation of the elastic fibers (orcein stain), without evidence of malignancy, findings consistent with temporal arteritis (Figure 5, Table 2). The salivary gland biopsy was unremarkable, which makes these diagnoses less likely but, given the limited sensitivity of this procedure, does not exclude them. Corticosteroids were then relayed with oral prednisone 60 mg/day (about 1 mg/kg/day), to be maintained for four weeks before a gradual taper, with routine potassium and calcium/vitamin D supplementation to prevent corticosteroid-related hypokalemia and hypocalcemia.

**Figure 5:**
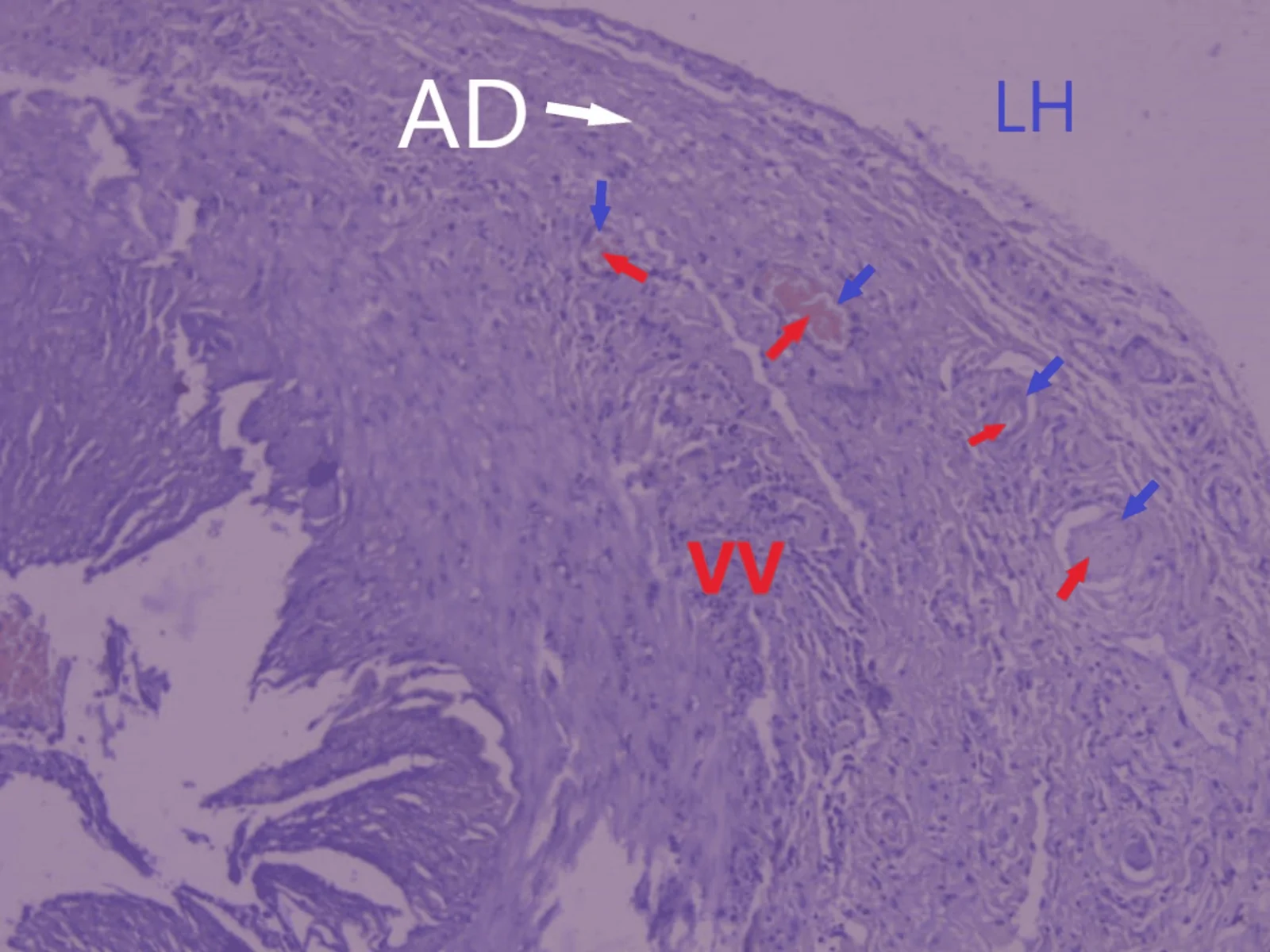
Temporal artery biopsy (hematoxylin-eosin) showing a dense lympho-histiocytic infiltrate with periadventitial cuffing around several vasa vasorum, predominating in the adventitia, consistent with temporal arteritis. AD: adventitia; VV: vasa vasorum (red arrows); LH: lympho-histiocytic infiltrate/cuffing (blue arrows).

She subsequently developed respiratory discomfort with an oxygen saturation of 96% on 4 L/min supplemental oxygen and bibasal crackles; a thoracic CT angiogram showed a non-cardiogenic (lesional) pulmonary edema with a small pleural effusion and no proximal pulmonary embolism (Figure 6), a picture considered by the treating team more consistent with a pulmonary inflammatory/vasculitic manifestation than with a distinct infection (no organism identified at that stage), although an early infectious process could not be formally excluded. She remained on oral prednisone 60 mg/day and received dual antibiotic therapy (third-generation cephalosporin 2 g/day and ciprofloxacin 400 mg twice daily) for five days, with clinical improvement.

**Figure 6:**
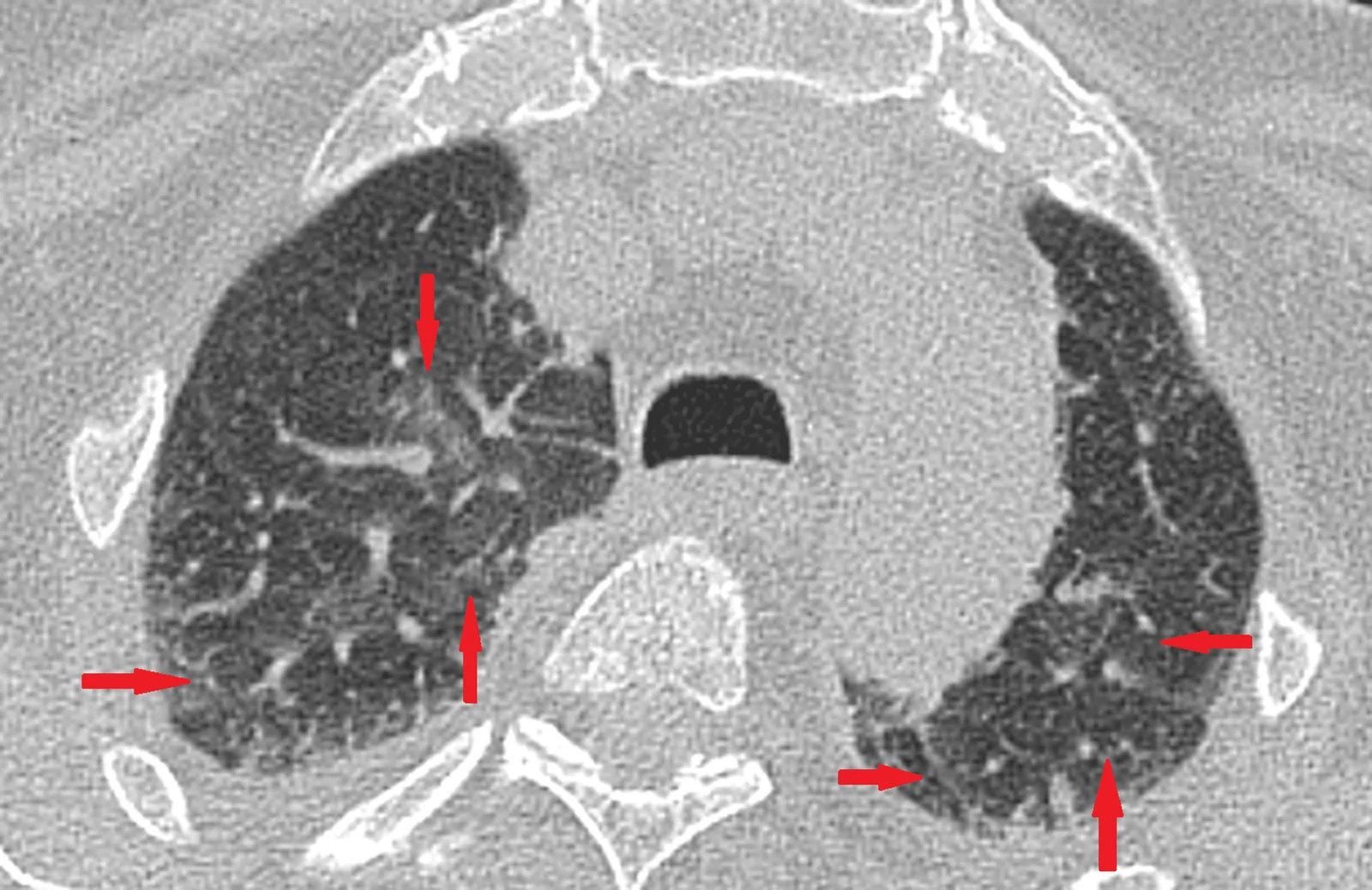
Axial lung-window chest CT showing bilateral ground-glass and reticular opacities (arrows), consistent with the non-cardiogenic (lesional) pulmonary edema described in the Case Presentation section.

An FDG-PET/CT scan was requested by the internal medicine team for disease-extent assessment and therapeutic orientation, but it could not ultimately be performed because of local resource unavailability. The patient remained on full-dose oral prednisone (60 mg/day).

She subsequently developed acute respiratory distress with paradoxical thoraco-abdominal breathing, an oxygen saturation of 93% on 12 L/min through a high-concentration mask, and a fever spiking to 41 °C, approximately three weeks after starting oral prednisone, still at 60 mg/day. Blood cultures (aerobic and anaerobic) grew Gram-positive cocci identified as a *Streptococcus* species (Table 2). Serum electrolytes obtained at that time showed severe hyponatremia (sodium 119 mmol/L) and severe hypokalemia (potassium 2.7 mmol/L), despite routine potassium supplementation, with a C-reactive protein of 8 mg/L; a procalcitonin was requested but could not be processed (Table 1). The last blood count, obtained in the intensive care unit, showed leukocytes 3.46 × 10⁹/L, lymphocytes 0.07 × 10⁹/L, and platelets 32 × 10⁹/L (Table 1). She was transferred to the intensive care unit of another department, where she died after a two-week stay in the context of septic shock; the details of organ support and terminal events were not available to the authors. The clinical timeline is summarized in Table 3.

**Table 3:** Timeline of clinical events FDG-PET/CT: fluorodeoxyglucose with positron emission tomography/computed tomography; GCA: giant cell arteritis; CRP: C-reactive protein; CSF: cerebrospinal fluid; MRI: magnetic resonance imaging; ESR: erythrocyte sedimentation rate; CRAO: central retinal artery occlusion

Time point	Event
Symptom onset 6 months before admission	Left lower-limb weakness and sciatica, interpreted as an L4–L5 disc herniation
About 3–4 months before admission	External lumbar MRI: multilevel disc-osteoarthritis, severe canal stenosis at L4–L5 and L5–S1, normal conus medullaris
After the lumbar MRI 2 months before admission	L4–L5 laminectomy; no improvement
Following months	Complete left lower-limb paralysis refractory to rehabilitation; new right lower-limb deficit with urinary retention (reason for hospitalization)
4 days before admission	Right upper-limb weakness, left ocular redness, and rapidly progressive left visual loss
Admission (day 0)	Myelopathic and right hemicerebellar syndromes; left CRAO on fundoscopy; CRP 56.9 mg/L, ESR 48 mm/h, procalcitonin 0.04 ng/mL; workup: CSF analysis, spinal and brain MRI, echocardiography, supra-aortic Doppler, CT angiography
Early hospitalization	Methylprednisolone 1 g/day for 5 days; improvement in right upper-limb strength; internal medicine consulted
Following days	Temporal artery and minor salivary gland biopsies, confirming GCA; relay with oral prednisone 60 mg/day (to be maintained for 4 weeks, then tapered) with potassium and calcium/vitamin D supplementation
Subsequently	Respiratory discomfort; non-cardiogenic pulmonary edema on thoracic CT; dual antibiotic therapy (third-generation cephalosporin and ciprofloxacin) for 5 days with improvement; prednisone continued.
Around the same time	FDG-PET/CT requested for disease-extent assessment, not performed (resource unavailability)
Later (about 3 weeks after starting prednisone)	Acute respiratory distress, fever 41 °C, *Streptococcus* bacteremia, sodium 119 mmol/L, potassium 2.7 mmol/L, CRP 8 mg/L, full-dose prednisone still ongoing
Outcome	Intensive care in another department for two weeks; death in septic shock

Written informed consent for publication of this case report and the accompanying clinical, radiological, and histological images was obtained from the patient's next of kin.

## Discussion

The inaugural myeloradiculitis was the most misleading feature of this observation. In a 61-year-old woman with unilateral leg weakness and sciatica and a lumbar MRI showing severe canal stenosis, a degenerative disc etiology was a reasonable first hypothesis, and she underwent laminectomy accordingly. The absence of headache, jaw claudication, and scalp tenderness - the classic hallmark of the disease [1] - meant that GCA was not considered until a contralateral deficit, a sensory level, and sphincter involvement emerged, by which time surgery had already been performed without benefit. Spinal cord and nerve root involvement in GCA remains a very rare, largely case-report-based entity with an uncertain mechanism; early descriptions attribute it to ischemia of the radicular arteries arising from an inflamed, ectatic aorta [2,3]. In our patient, the partial intrathecal immunoglobulin synthesis identified on CSF oligoclonal band analysis raises the possibility of an inflammatory component, since acute ischemic events do not typically generate intrathecal antibody synthesis [6]; it does not, however, prove an inflammatory myeloradiculitis or exclude spinal ischemia, and no spinal tissue was available for histological confirmation. This case suggests that a treatment-refractory myelopathy in an older patient with systemic inflammatory features may warrant reassessment for an underlying vasculitic process, since disc pathology and vasculitic myeloradiculitis can be difficult to distinguish clinically.

The multifocal cerebral involvement - a right hemicerebellar syndrome together with FLAIR lesions in the frontal, parietal, occipital, cerebellar, and bulbo-cervical regions - is compatible with the extension of large-vessel disease to the supra-aortic trunks, a phenomenon detected by CT angiography in 27 of 40 patients (67.5%) with newly diagnosed, biopsy-proven GCA in a prospective study [7]; here it was accompanied by a complete occlusion of the right internal carotid artery and an ectatic ascending aorta. The inter-arm systolic blood pressure difference noted on admission (104 mmHg on the left versus 92 mmHg on the right, a 12 mmHg difference) is a simple, often underused bedside sign of proximal large-vessel stenosis (subclavian or axillary artery) in this disease, and was consistent with - though clinically more subtle than - the large-vessel involvement subsequently confirmed on imaging. GCA-related cerebrovascular ischemic events remain uncommon, occurring in a small percentage of patients overall, but they cluster around the time of diagnosis and carry disproportionate morbidity; recognized risk factors include older age, smoking, and ischemic ophthalmic involvement [8,9]. In this patient, she had no smoking history, but the presence of ischemic ophthalmic involvement (the CRAO) was consistent with this recognized risk profile.

The acute left-eye visual loss in this case was due to a CRAO rather than the more classically described arteritic anterior ischemic optic neuropathy (AAION), reflecting occlusion of the central retinal artery rather than the posterior ciliary circulation supplying the optic nerve head. Notably, the left internal carotid artery was patent on CT angiography, indicating that the left CRAO arose from a more distal, branch-level occlusive process (the ophthalmic artery or its central retinal/ciliary branches) rather than from proximal carotid disease; taken together with the contralateral (right) proximal internal carotid occlusion, this illustrates the multifocal nature of the vasculitic process, affecting different vascular territories at different levels on each side. Both entities share a common arteritic mechanism, and both mandate urgent high-dose corticosteroid therapy, chiefly to protect the contralateral eye, since established visual loss is rarely reversible in either condition [10,11].

Given the multifocal, atypical presentation, several differential diagnoses required exclusion. Oligoclonal band analysis of the CSF and serum showed a type 3 pattern - identical bands present in both CSF and serum, together with a small number of additional CSF-restricted bands - indicating a combination of systemic immune activation and a limited degree of intrathecal immunoglobulin synthesis; this pattern is more in keeping with a systemic inflammatory or vasculitic process than with an isolated, primary central nervous system inflammatory or demyelinating disease. This, together with negative ANCA and anti-double-stranded DNA serologies, negative CSF tuberculosis and Lyme testing, negative syphilis and HIV serologies, and a normal serum ACE, argued against the main alternative diagnoses and supported a systemic vasculitic mechanism instead. The minor salivary gland biopsy was performed to screen for granulomatous (sarcoidosis) and lymphoproliferative mimics; it was unremarkable, which makes these diagnoses less likely but, given its limited sensitivity, does not exclude them. The diagnosis of GCA rested on the temporal artery histology, which showed a lympho-histiocytic infiltrate with periadventitial cuffing of the vasa vasorum.

In this case, FDG-PET/CT - increasingly used to map the extent of large-vessel involvement and to guide the choice and duration of glucocorticoid-sparing therapy - could not be performed because of local resource constraints. This is a common real-world limitation, particularly outside specialized centers, and current EULAR imaging recommendations acknowledge that CT angiography, MR angiography, and ultrasound remain acceptable alternatives for assessing large-vessel involvement when FDG-PET/CT is not readily available [12]. In this patient, the diagnosis was established on temporal artery biopsy and large-vessel involvement was documented by CT angiography and echocardiography, although its full extent could not be assessed without FDG-PET/CT; urgent treatment was therefore not delayed pending PET-CT.

The fatal outcome illustrates the infectious vulnerability of patients with GCA under high-dose corticosteroid therapy; here, the septic episode occurred approximately three weeks after starting oral prednisone, while she was still receiving the full 60 mg/day dose (the planned four-week full-dose period had not ended and no taper had begun), in addition to the five days of intravenous methylprednisolone pulses given earlier. The earlier non-cardiogenic pulmonary edema was considered more consistent with an inflammatory/vasculitic manifestation than with infection, but an early infectious process cannot be excluded: the admission procalcitonin does not apply to this later episode, and she improved under antibiotics and full-dose corticosteroids given together. The subsequent Streptococcus bacteremia developed later in the hospital course without an identified preceding focus. GCA itself is associated with an increased risk of severe infection and infection-related mortality during the first year after diagnosis, a risk that rises further with cumulative glucocorticoid exposure [13,14]. The severe hyponatremia and hypokalemia appeared only during the septic episode, despite routine potassium supplementation; their mechanism could not be elucidated, as intensive care data were not available to the authors.

Glucocorticoid-sparing biologic therapy with tocilizumab is now endorsed for severe or refractory forms of large-vessel GCA by the EULAR recommendations for the management of large-vessel vasculitis, on the basis of the randomized GiACTA trial and its long-term extension, both because of its direct anti-inflammatory effect and because it allows a faster glucocorticoid taper [15-17]. In this patient, tocilizumab was not used, and whether earlier glucocorticoid-sparing therapy would have changed the outcome cannot be determined.

This report has several limitations. The full extent of large-vessel involvement could not be mapped with FDG-PET/CT, which was unavailable; the diagnosis and treatment decisions nonetheless rested on converging evidence from temporal artery biopsy, CT angiography, and echocardiography. No electrophysiological study was performed before admission, no spinal tissue was available, and the lumbar MRI was performed externally, with only the radiology report available; MRI findings other than those illustrated are reported from the radiology reports. The causative *Streptococcus* isolate was not identified to the species level, a procalcitonin result was not available during the septic episode, and, as the patient was transferred to intensive care in another department, details of organ support and terminal events were not available, limiting full characterization of the terminal septic episode. Finally, as with any single case report, the generalizability of the described disease course and of the practical lessons drawn from it is inherently limited.

## Conclusions

This case highlights four practical lessons. First, in this patient, a treatment-refractory myelopathy accompanied by a systemic inflammatory syndrome was ultimately explained by GCA; in older patients with such a picture, even without headache, jaw claudication, or scalp tenderness, vasculitis may be worth considering, since a presumed disc pathology can otherwise mask it and lead to unhelpful surgery. Second, corticosteroid therapy should be initiated without delay whenever GCA is clinically suspected, given the risk of irreversible ischemic progression, and should not be withheld pending histological or imaging confirmation. Third, here, treatment was started on the basis of clinical assessment, temporal artery biopsy, and conventional vascular imaging, without waiting for FDG-PET/CT. Fourth, close infection surveillance and consideration of guideline-supported glucocorticoid-sparing treatment may be appropriate in selected patients with severe GCA.

This report adds to the limited literature on spinal-onset GCA with multifocal ischemic complications.
